# The effects of different surgical positions (semi-sitting and lateral position) on the surgical outcomes of large vestibular schwannoma: study protocol for a randomized controlled trial

**DOI:** 10.1186/s13063-022-06437-z

**Published:** 2022-06-14

**Authors:** Xiaolong Wu, Xu Wang, Gang Song, Mingchu Li, Chengbei Hou, Ge Chen, Hongchuan Guo, Xinru Xiao, Jie Tang, Qingtang Lin, Yuhai Bao, Jiantao Liang

**Affiliations:** 1 Department of Neurosurgery, Xuanwu Hospital, Capital Medical University, Beijing, China; 2 International Neuroscience Institute (China-INI), Beijing, China; 3 Centre for Evidence-Based Medicine, Xuanwu Hospital, Beijing, China

**Keywords:** Percentage of gross total resection, Facial nerve function, Randomized controlled trial, Study protocol, Surgical position, Vestibular schwannoma

## Abstract

**Background:**

There is an ongoing discussion about the advantages and disadvantages of different surgical positions (semi-sitting and lateral position) for vestibular schwannoma surgery. Each position has its advantages, disadvantages, challenges, and risk profiles. The objectives of this study are to compare the effects of different surgical positions (semi-sitting and lateral position) on the outcomes of large vestibular schwannoma, primarily including effectiveness and safety.

**Methods:**

In this single-centre, open, randomized controlled trial, we will recruit a total of 116 participants according to the inclusion and exclusion criteria who will be randomized to an experimental group or control group. Patients will undergo operations in semi-sitting and lateral positions. The primary endpoint will be the percentage of gross total resection. The secondary endpoints will include the facial nerve function, hearing preservation, surgical position placement time, time of operation (skin-to-skin surgical time), hospital stay, total hospitalization fee, and complications. The follow-up period will be at least 12 months, during which time patients will be evaluated both clinically and radiologically.

**Discussion:**

This issue is still debated after 30 years since the first large comparative study was published in 1989, so the study will be useful. Therefore, more high-quality studies are required to compare clinical outcomes, complications, and other factors associated with these two positions.

**Trial registration:**

Chinese Clinical Trial Registry ChiCTR1900027550. Registered on 17 November 2019

## Administrative information

**Table:** 

Title {1}	The effects of different surgical positions (semi-sitting and lateral position) on the surgical outcomes of large vestibular schwannoma: study protocol for a randomized controlled trial
Trial registration {2a and 2b}.	The study has been registered in the Chinese Clinical Trial Registry (ChiCTR1900027550, http://www.chictr.org.cn/edit.aspx?pid=45738&htm=4) on 17th Nov 2019.
Protocol version {3}	V1.1, Version Date: October 11, 2019
Funding {4}	None
Author details {5a}	Xuanwu Hospital, Capital Medical University, Beijing, China
Name and contact information for the trial sponsor {5b}	None
Role of sponsor {5c}	None

## Introduction

### Background and rationale {6a}

Vestibular schwannoma (VS) is a common intracranial space-occupying lesion, accounting for approximately 6–10% of intracranial tumours. VS most often develops from the vestibular nerve of the internal auditory canal segment. Its origin is the Schwann cell cover of the eighth cranial nerve. Early auditory disorders, such as hearing deterioration and tinnitus, are common first symptoms [1–5]. However, space exists in the cerebellopontine area (CPA), and some patients have large tumours without any symptoms. Although VS exhibits a benign pathologically, tumours can eventually become life-threatening as brainstem compression increases. Regarding treatment, controversy still exists, but the current strategy depends substantially on the tumour size. Generally, for small tumours, conservative management or radiosurgery can be used, whereas surgery is inevitable in large or growing tumours [5–9]. However, some scholars have noted that tumour removal should be performed at the earliest stage as much as possible, especially for large tumours (Koos 4) accompanied by obvious brainstem and cerebellum compression [10–12].

**Fig. 1:**
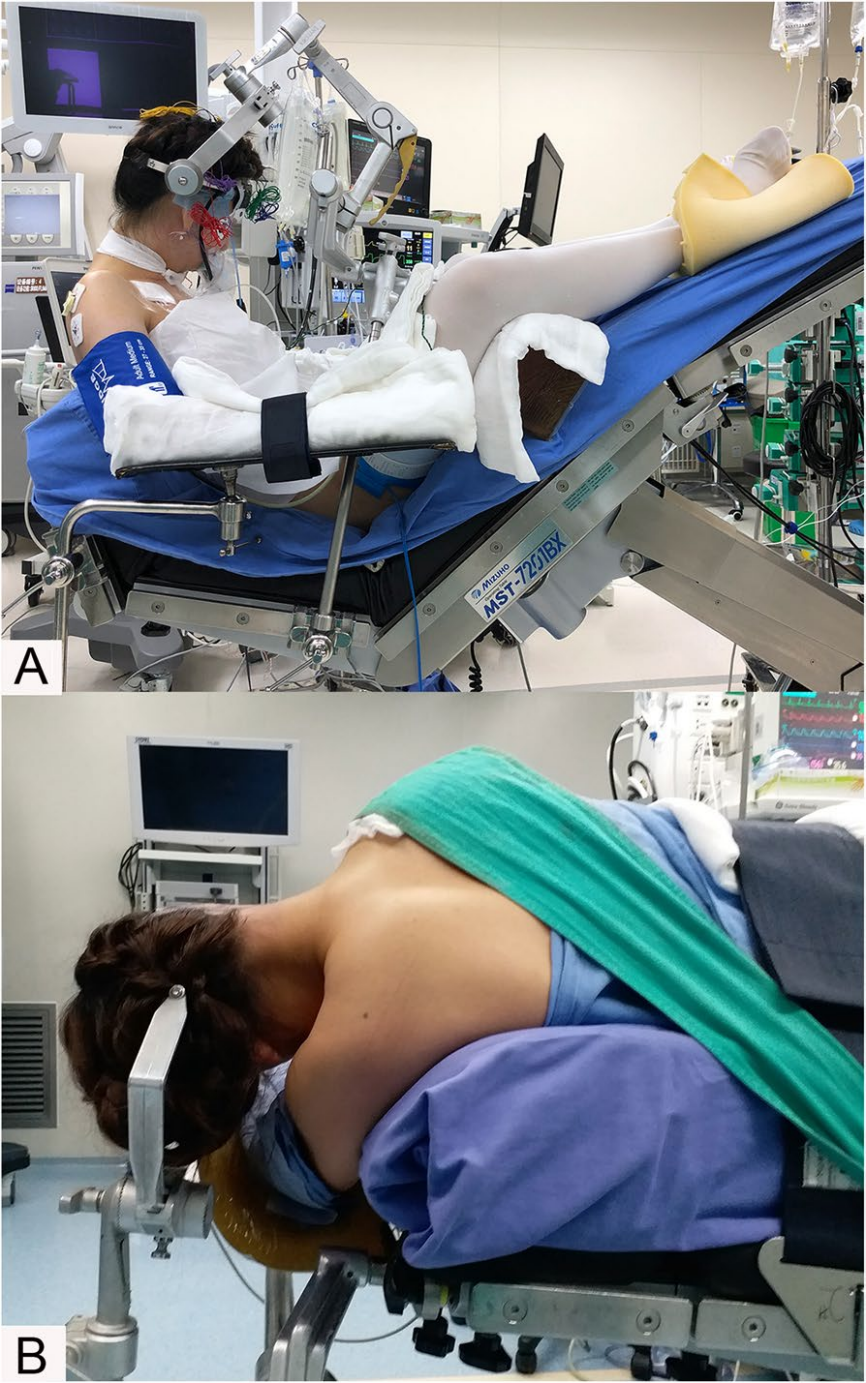
Semi-sitting position (**A**) and lateral position (**B**)

The goals of modern VS surgery are to completely remove the tumour, preserve nerve function, and reduce complications. In recent years, with the popularization and application of microscopy, continuous improvements in surgical instruments and skills, in-depth studies of the microanatomy of the cerebellopontine angle, and the application of intraoperative electrophysiological monitoring technology, the above surgical goals have largely been attained [12–15]. In addition, many factors affect the outcomes of surgery and related complications, among which the effect of different surgical positions has long been debated. Patients are easily placed in the lateral position (LP, Fig. 1B), and few complications are associated with this surgical position. However, corresponding disadvantages do exist, such as high cranial pressure, difficulty in releasing cerebrospinal fluid, unclear anatomical location, and increased requirements for assistants [5]. In contrast, the semi-sitting position (SSP, Fig. 1A) provides specific possible advantages, including drainage of blood and cerebrospinal fluid (CSF) by gravity, decreased bleeding, clean surgical field, improved surgical exposure and anatomical orientation, and ability to perform continuous bimanual dissection [5, 15–18]. However, this position carries some potential risks, such as venous air embolism (VAE) [19], tension pneumocephalus, and peripheral nerve injury [16, 20]. Despite these risks, some neurosurgeons preferred to perform posterior fossa surgeries in the SSP [15, 21]. Many studies have shown that the operational risk associated with body position is very low in the context of adequate preoperative preparation, intraoperative coordination with anaesthesiologists, and careful monitoring. Thus, SSP is safe and reliable [22–24]. Professor Samii of Hannover, Germany, personally completed more than 4000 cases of semi-sitting VS resection. Long-term functional outcomes and surgery-related complication rates were satisfactory. Fifty patients with VSs > 4.0 cm in maximal extra-meatal diameter were reported with gross total resection, and the anatomical integrity of the facial nerve was preserved in 92% [25]. Taking all the above-mentioned advantages into consideration, SSP may lead to better surgical outcomes, such as a higher percentage of gross total resection and better facial nerve function, for patients with large VSs. However, randomized studies are required to verify this hypothesis.

Literature searches were performed in August 2019 and then updated in February 2020 using the PubMed database. We identified a few studies that reported the surgical results of SSP compared with LP on the VS, including a randomized, multicentre trial and a few retrospective studies. These results and conclusions of different studies are quite different, and the evaluation indexes in various studies are relatively limited. Further comprehensive and in-depth research is needed. We also conducted a comprehensive search on the ClinicalTrials.gov and Chinese Clinical Trial Registry and found no similar prospective randomized controlled trials (RCTs) that are ongoing or completed.

### Objectives {7}

The objectives of this study are to compare the effects of different surgical positions (SSP and LP) on the outcomes of large VS, primarily including effectiveness and safety.

### Trial design {8}

This trial will be designed as a single-centre, open, RCT to assess the effects of different surgical positions (SSP and LP) on the surgical outcomes of large VS. The protocol was reported using the recommendations of Standard Protocol Items: Recommendations for Interventional Trials (SPIRIT) [26]. The open mode is the only possible choice for this trial because it is impossible to hide the surgical position of each patient. However, to minimize bias related to the “open” mode, the evaluation of some outcomes and the statistical analysis will be performed in a blinded fashion. The schedule of enrolment, interventions, and assessments for all study patients can be found in Fig. 3.

## Methods: participants, interventions, and outcomes

### Study setting {9}

The clinical trial will be conducted at a tertiary referral neurotological centre. The research team included neurosurgeons, anaesthetists, and statisticians. The details of the investigators and research sites are provided in Table 1.

**Table 1:** Investigators and research sites of the study

Role	Name	Specialty	Research site
**Principal investigator**	Jiantao Liang	Neurosurgeon	Xuanwu Hospital, Capital Medical University
**Associate investigator**	Yuhai Bao	Neurosurgeon	Xuanwu Hospital, Capital Medical University
	Lei Zhao	Anaesthetist	Xuanwu Hospital, Capital Medical University
	Ting Ma	Anaesthetist	Xuanwu Hospital, Capital Medical University
	Qinghai Liu	Anaesthetist	Xuanwu Hospital, Capital Medical University
	Gang Song	Neurosurgeon	Xuanwu Hospital, Capital Medical University
	Xu Wang	Neurosurgeon	Xuanwu Hospital, Capital Medical University
	Xiaolong Wu	Neurosurgeon	Xuanwu Hospital, Capital Medical University
**Data management statistician**	Chengbei Hou	Statistician	Xuanwu Hospital, Capital Medical University

### Eligibility criteria {10}

**Fig. 2:**
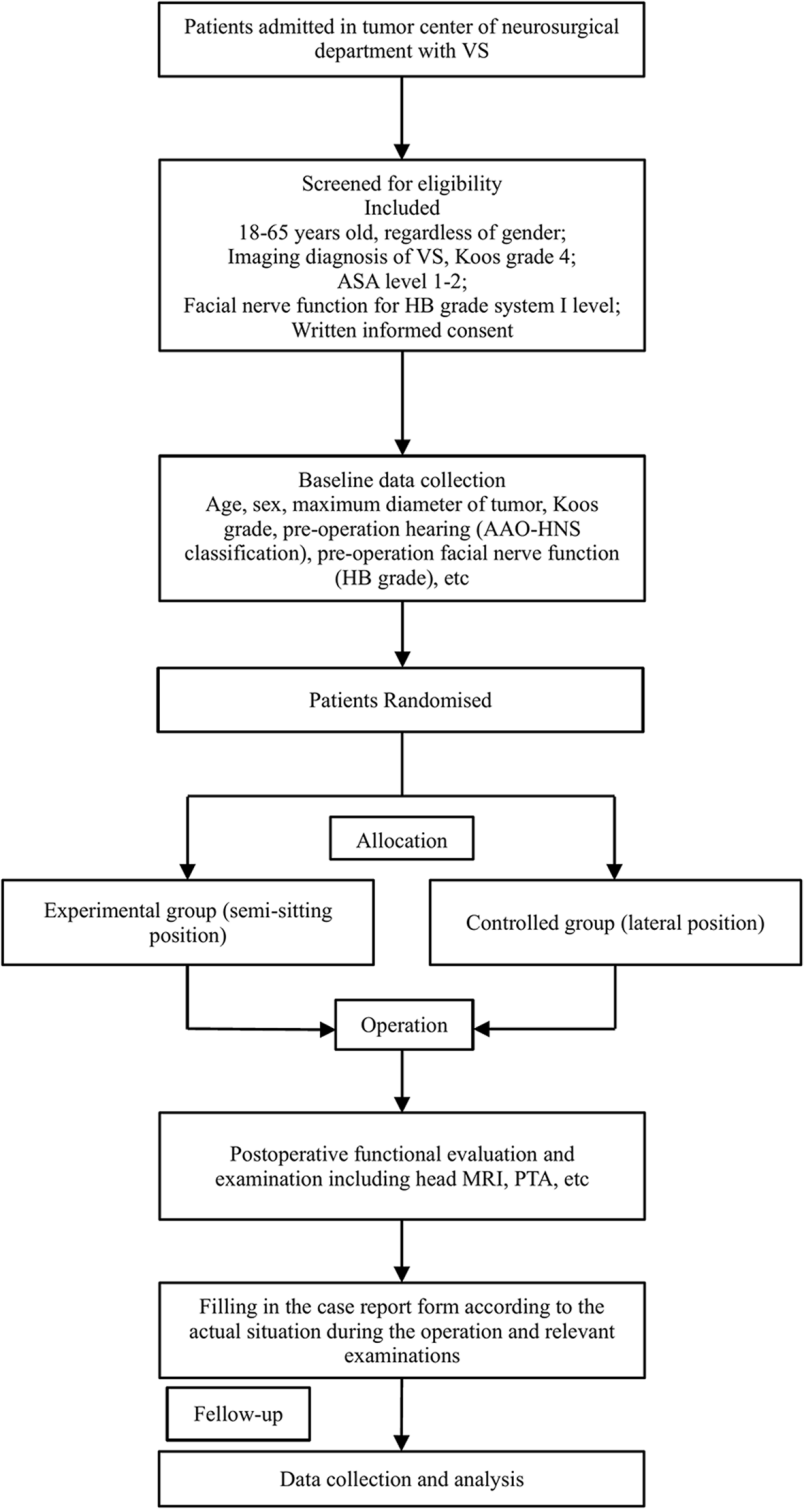
Flow diagram of this single-centre, open, randomized controlled trial

Participant recruitment will be performed at our tertiary referral neurotological centre. Patients with VSs who fulfil the inclusion criteria and sign the informed consent forms will enter the screening period. Participants who meet the exclusion criteria will be excluded before randomization. Figure 2 shows the flow chart of the study. In this trial, patients who met the inclusion and exclusion criteria will be randomly assigned to two parallel groups, namely, an experimental group or a control group, and the baseline data of each patient will be collected carefully. The details of the inclusion and exclusion criteria and withdrawal criteria are provided in Table 2.

**Table 2:** Inclusion and exclusion criteria and withdrawal criteria

**Inclusion criteria**	
Age between 18 and 65 years old, regardless of gender	
Preoperative imaging diagnosis of VS, Koos grade 4	
Preoperative American Society of Anaesthesiologists (ASA) score 1–2 [27, 28]	
Preoperative evaluation of facial nerve function for House-Brackmann (HB) grading system I level [29]	
**Exclusion criteria**	
History of any forms of therapies, such as radiotherapy and operation	
Cervical spondylolisthesis and cervical instability	
Diagnosis of neurofibromatosis type 2 (NF2)	
**Withdrawal criteria**	
Patient wishing to interrupt participation in the study before the end	
Patient has a patent foramen ovale^a,b^	
Postoperative pathological diagnosis is non-schwannoma	

*VS* Vestibular schwannoma

^a^Screening method for the preoperative detection of a patent foramen ovale (PFO) for patients with planned SSP is transoesophageal echocardiography (TEE)

^b^If the patient has a PFO, he or she will not be excluded from the study, but the surgical position originally assigned will be changed to LP

### Additional consent provisions for collection and use of participant data and biological specimens {26b}

Not applicable as no biological specimens were collected as part of this trial.

### Interventions

#### Explanation for the choice of comparators {6b}

The details are provided in Table 3.

**Table 3:** Primary and secondary outcomes

**Primary outcome**		
Percentage of gross-total resection		
Extent of tumour resection	Gross total resection: total resection under the microscope, imaging without residual.	Note: The evaluation of total resection under the microscope and determination of microscopic residual tumours depend largely on subjective observations from experienced neurosurgeons according to the operation video.
	Near-total resection: microscopic residual (a few tumour capsules, < 5%), imaging no residual.	
	Subtotal resection: microscopic residual (small tumour remnant, 5–10%), imaging small tumour remnant residual.	
	Partial resection: microscopic nodular residual tumour (≥ 10%), imaging nodular residual tumour.	
**Secondary outcomes**		
Facial nerve function	As one of the inclusion criteria, facial nerve function was documented photographically at rest and while performing standardized facial expressions at defined time points (preoperatively, discharge, and 6 and 12 months after surgery).	This feature was evaluated by two experienced neurologists and classified according to the HB grading system [29].
Hearing function	Hearing level will be analysed and evaluated using pure-tone audiometry (PTA) and speech discrimination score (SDS) according to the guidelines of the American Association of Otolaryngology–Head and Neck Surgery (AAO-HNS) classification [30].	Serviceable hearing was defined as either class A (PTA ≤ 30 dB, SDS ≥ 70%) or class B (30 < PTA ≤ 50 dB, SDS ≥ 50%) and non-serviceable hearing as class C (PTA > 50 dB, SDS ≥ 50%) or class D (any PTA, SDS < 50%).
Surgical positioning time		
Time of operation	Craniotomy time	
	Intradural microsurgery time	
	Scalp closure surgery time	
Hospital stay		
Total hospitalization fee		
General complications	Intracranial haematoma: head imaging examination will be performed within 3 h after surgery.	
	Cerebrospinal fluid leakage (incision leakage or nasal leakage).	
	Intracranial infection: postoperative fever, cerebrospinal fluid routine, and biochemical evidence of infection.	
	Cranial nerve disorders in the posterior group: drinking water choking cough, articulation disorder, etc.	
	Others	
Special complications	VAE	
	Others	
Recurrence		

*HB* House-Brackmann, *VAE* Venous air embolism

#### Intervention description {11a}

All surgeries will be performed via the retrosigmoid approach with intraoperative neurophysiological monitoring (brainstem auditory evoked potentials, continuous facial nerve electromyography, and direct facial nerve stimulation). All surgeries will be performed by two experienced neurosurgeons (J.T.L. and Y.H.B.). Both surgeons will operate on patients in the SSP and LP. Before this clinical trial, they performed at least 200 VS surgeries with SSP and LP, respectively. They have no preference regarding the SSP and LP and are skilled in operating under both positions. Both surgeons will operate on patients in the SSP and LP. The LP adopted by the control group is a conventional position for neurosurgery, while the SSP is adopted by the experimental group, which is similar to that reported in the Frankfurt Protocol [23].

#### Criteria for discontinuing or modifying allocated interventions {11b}

The details are provided in Table 1.

#### Strategies to improve adherence to interventions {11c}

Not applicable as the interventions are all surgery.

#### Relevant concomitant care permitted or prohibited during the trial {11d}

Relevant concomitant care, such as hypertension and diabetes, is permitted during the trial.

#### Provisions for post-trial care {30}

Even after the clinical trial is finished, a long-term follow-up will be achieved in each patient.

### Outcomes {12}

The primary endpoint will be the percentage of gross total resection. The secondary endpoints will include facial nerve function, hearing preservation, surgical position placement time, time of operation (skin-to-skin surgical time), hospital stay, total hospitalization fee, and complications. The details of the primary and secondary outcomes are provided in Table 3.

#### Participant timeline {13}

**Fig. 3:**
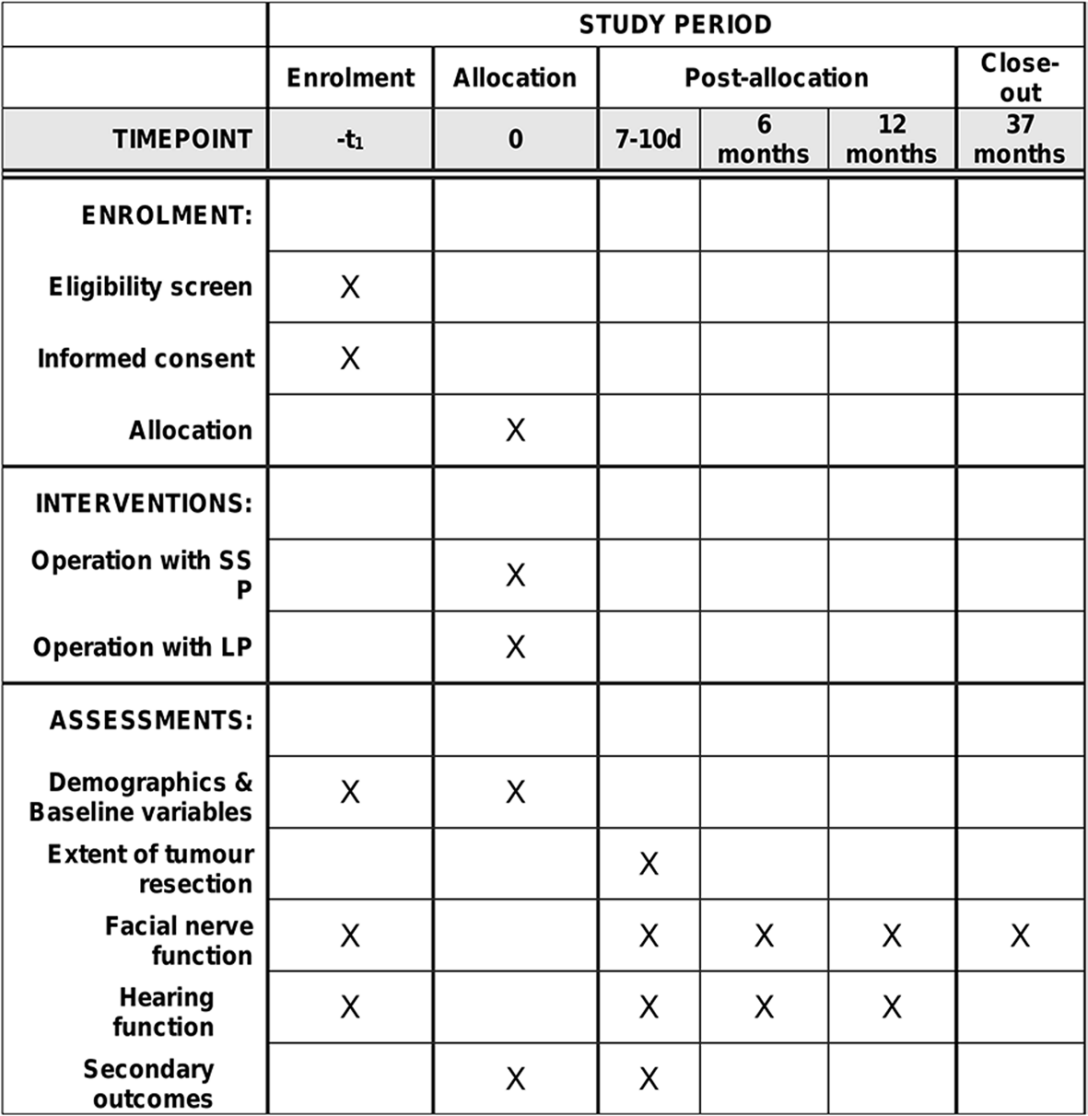
Schedule of enrolment, interventions, and assessments for all study patients

The timeline is shown in Fig. 3.

#### Sample size {14}

The sample size was calculated from the primary endpoint. We calculated the sample size as of September 2019 based on the results of the only multicentre randomized controlled trial in which the percentage of gross total resection in the SSP and LP were 93% and 73%, respectively [14]. The sample size calculated by PASS version 15 is 58 participants per group (overall sample = 116 participants) according to the above data with a power of 80%, an alpha risk of 5%, and an estimated shedding rate of 8%.

#### Recruitment {15}

Participant recruitment will be performed at the tertiary referral neurotological centre.

### Assignment of interventions: allocation

#### Sequence generation {16a}, concealment mechanism {16b}, and implementation {16c}

Randomized lists will be created before the beginning of the study by SPSS version 21. Then, several sealed and opaque envelopes will be made and kept securely by recognized third parties. To ensure the balance of the number of patients and age distribution between the two groups, we considered age stratification (18–35 years old and 36–65 years old) with an appropriate block size. After the patient is enrolled, the sealed envelope system will be used for the randomization of the patients into two groups. Neither the investigator nor the participants will be aware of each envelope’s contents.

### Assignment of interventions: blinding

#### Who will be blinded {17a}

Doctors and patients involved in the study will be aware of the group allocations. However, the evaluation of primary and secondary outcomes and statistical analysis will be entrusted to a third party that is not involved in the study. All patients will be identifiable with a unique study number. During the evaluation process, all members of this third party will be blind to the participant’s allocation.

#### Procedure for unblinding if needed {17b}

Not applicable because doctors and patients involved in the study will be aware of the group allocations.

### Data collection and management

#### Plans for assessment and collection of outcomes {18a} and data management {19}

The data in the case report form (CRF) provided by researchers should be accurate, complete, timely, and reliable. Baseline data of participants (age, sex, tumour maximum diameter, hearing level, and facial nerve function level) will be collected when they are enrolled. Independent data administrators not involved in the treatment will collect and aggregate the individual data using an online electronic data system.

#### Plans to promote participant retention and complete follow-up {18b}

The follow-up period will be at least 1 year, during which time the patients will be evaluated both clinically and radiologically. After surgery, all patients will be regularly examined at the outpatient clinic every 3–6 months. Clinical assessments, such as facial nerve function (according to the HB grading system) and hearing function (according to the AAO-HNS classification), will be performed. Postoperative MRI will be performed before discharge and 3 months after surgery. Further follow-up examinations will be performed every year. No interim analyses are planned.

#### Confidentiality {27}

The main researchers should be qualified based on the “Good Clinical Practice (GCP) training” of the State Food and Drug Administration for compliance with the training. All investigators will make every effort to ensure the safety of patient information. An independent clinical monitor within our hospital will perform an annual monitoring assessment. The main auditing procedures will include ensuring that informed consent has been obtained from all the included patients based on regulatory guidelines and verifying the content of the case report forms.

#### Plans for collection, laboratory evaluation, and storage of biological specimens for genetic or molecular analysis in this trial/future use {33}

Not applicable as no biological specimens were collected as part of this trial.

## Statistical methods

### Statistical methods for primary and secondary outcomes {20a}

All analyses will be performed using the SPSS software, version 25 (IBM Corp.) with a two-sided significance level of .05 unless otherwise stated. Continuous quantitative variables are expressed as numbers, means, standard deviations, and medians. Comparisons between proportions for the categorical variables will be performed using the chi-square test, Fisher’s exact test, or Mann-Whitney *U* test. In addition, Student’s *t* test and non-parametric analysis will be used for continuous variables with and without normal distributions. Differences in the percentage of gross total resection, facial nerve function, hearing function, general complications, and special complications between the SSP and LP groups will be evaluated using Fisher’s exact test or chi-squared test. Differences in the surgical positioning time, time of operation, hospital stay, and total hospitalization fee between the SSP and LP groups will be evaluated using Student’s *t* test or Mann-Whitney *U* test. Regression analysis will be used to evaluate the influence of SSP versus LP, tumour size, and surgeons on the percentage of gross total resection, as well as SSP versus LP, tumour size, surgeons, and the percentage of gross total resection on facial nerve function and hearing function. Association of the time of operation with SSP versus LP, tumour size, the percentage of gross total resection, and surgeons will be evaluated using ANOVA. If the patient has a PFO, he or she will not be excluded from the study but analysed according to the SSP group they were originally assigned after the LP surgery.

### Interim analyses {21b}

An interim analysis is planned when the 12-month follow-up data of the first 50 randomized patients are obtained. Stopping guidelines are not applicable.

### Methods for additional analyses (e.g. subgroup analyses) {20b}

Not applicable as we have made this clear in the “Statistical methods for primary and secondary outcomes {20a}” section.

### Methods in analysis to handle protocol non-adherence and any statistical methods to handle missing data {20c}

Data collection and follow-up will be promoted by scheduling every foreseen follow-up moment (7–10 days, 6 months, and 12 months postoperatively) in advance. In the case of drop-out, the reason for drop-out or “unknown reason for drop-out” will be logged qualitatively. Missing or incorrect data will be detected by software programs and will be reported transparently in the publication of trial results.

### Plans to give access to the full protocol, participant-level data, and statistical code {31c}

There are no data associated with the current paper, which describes a protocol for a clinical trial that is in progress at the time of submission. All investigators will have access to the final dataset. The datasets of the current clinical trial will be available from the corresponding author on reasonable request, and no personal details will be involved. The obtained results will be communicated to participants, clinicians, and the public through a journal article. All the participants’ personal information will be confidential to the public and journal.

### Oversight and monitoring

#### Composition of the coordinating centre and trial steering committee {5d}

It is supervised and coordinated by the Ethics Committee of Xuanwu Hospital.

#### Composition of the data monitoring committee, its role, and reporting structure {21a}

The data monitoring committee will consist of one neurosurgeon and two neurologists independent from this trial. If any modification exists, the changes should be submitted to the ethics committee and the trial approval department. All modifications will not be made without approval.

#### Adverse event reporting and harms {22}

Adverse events and postoperative complications will be assessed by the surgeon. If present, these adverse events and postoperative complications will be logged.

#### Frequency and plans for auditing trial conduct {23}

A yearly audit will be organized by the Ethics Committee. The main auditing procedures will include ensuring that informed consent has been obtained from all the included patients based on regulatory guidelines and verifying the content of the case report forms.

#### Plans for communicating important protocol amendments to relevant parties (e.g. trial participants, ethical committees) {25}

All important protocol amendments will be communicated by e-mail to the Ethical Committee.

#### Dissemination plans {31a}

If possible, all results from this clinical trial will be published in international peer-reviewed scientific journals or shown at the national conference and discussed with specialists in the relevant research field, regardless of whether the results are considered positive, negative, or inconclusive.

## Discussion

This trial is designed as a single-centre, open, RCT. The completion of this trial will provide a basis for patients with large VSs to choose more favourable and personalized surgical positions.

Each position has its own advantages, disadvantages, challenges, and risk profiles. The SSP offers both advantages and potentially serious risks, for example, VAE. The difference between SSP and LP surgery for the treatment of large VS remains controversial, and no randomized trials have been reported in meta-analyses and systematic reviews. As of February 2020, only one randomized, multicentre trial, a few single-centre, retrospective studies, and one review article have directly compared clinical outcomes and complications across the SSP and LP in VS surgery [5, 14, 15, 21, 31]. Scheller et al. [14] reported an increased rate of complete removal and improved facial nerve function after retrosigmoid VS surgery with SSP compared with LP. Roessler et al. [15] reported that patients with VSs operated in the SSP exhibit significantly better facial and cochlear nerve function postoperatively compared with LP without differences in complication rates. Spektor et al. [31] stressed that facial nerve preservation was significantly associated with the extent of tumour resection rather than surgical position in VS surgery. A systematic review [21] included 2 nonrandomized comparative studies and 8 noncomparative case series to compare the complications of VS surgery via the suboccipital retrosigmoid approach in the SSP versus LP. Safdarian et al. [21] reported no significant difference in outcome and safety between SSP and LP surgery according to the available evidence. Therefore, more high-quality studies are required to compare clinical outcomes, complications, and other factors for these two positions.

The study was designed to compare the effects of different surgical positions (SSP and LP) on the outcomes of large VS. We expect to improve knowledge of the effects of different surgical positions (SSP and LP) on the outcomes of large VS. The results from this trial will provide evidence for the selection of more favourable and personalized surgical positions for patients with large VSs.

## Trial status

At the time of manuscript submission, the status of the trial is recruiting. Originally, the expected duration of this study was 25 months from October 2019 to December 2021, and the recruitment duration was expected to last for 13 months from December 2019 to December 2020. However, as a result of the significant disruption that is being caused by the COVID-19 pandemic, participant recruitment was suspended for at least 5 months. After detailed discussions, we have decided that the participant recruitment period would be increased to 25 months from December 2019 to December 2021. The study will end after the last follow-up assessment 1 year later in December 2022.

## Data Availability

There are no data associated with the current paper, which describes a protocol for a clinical trial that is in progress at the time of submission.

## Abbreviations

AAO-HNS: American Association of Otolaryngology–Head and Neck Surgery
ASA: American Society of Anaesthesiologists
CPA: Cerebellopontine area
CRF: Case report form
CSF: Cerebrospinal fluid
COVID-19: Coronavirus disease-19
HB: House-Brackmann
LP: Lateral position
NF2: Neurofibromatosis type 2
PTA: Pure-tone audiometry
RCT: Randomized controlled trial
SDS: Speech discrimination score
SPIRIT: Standard Protocol Items: Recommendations for Interventional Trials
SSP: Semi-sitting position
TEE: Transoesophageal echocardiography
VAE: Venous air embolism
VS: Vestibular schwannoma
